# Electronic Nicotine Delivery Systems (ENDS) and Their Relevance in Oral Health

**DOI:** 10.3390/toxics7040061

**Published:** 2019-12-06

**Authors:** Gozde Isik Andrikopoulos, Konstantinos Farsalinos, Konstantinos Poulas

**Affiliations:** 1 Department of Pharmacy, University of Patras, Rio, 26500 Patras, Greece; gozdeisik85@yahoo.com (G.I.A.); kfarsalinos@gmail.com (K.F.); 2 Department of Cardiology, Onassis Cardiac Surgery Center, 17674 Kallithea, Greece; 3 National School of Public Health, Leof. Alexandras 196, 111521 Athens, Greece; 4 Institute of Research and Innovation NONSMOKE TEAM, Patras Science Park, Stadiou, Platani, Rio, 26504 Patras, Greece

**Keywords:** electronic cigarettes, periodontal health, oral health, periodontal disease, smoking, vaping

## Abstract

The number and popularity of electronic nicotine delivery systems (ENDS) and especially e-cigarettes (e-cigs) have been increasing in the last decade. Although ENDS owe their popularity to excluding the harmful chemicals that are present in tobacco smoke, there is a debate whether they are safe, regulated, and as harmless as they are assumed to be and have potential unknown long-term effects. Involvement of cigarette smoking to the progression of periodontal diseases, other adverse oral health outcomes, and its detrimental effects to oral health are well-described. ENDS producer companies claim that these products can improve oral health by providing alternatives to smoking. However, the effect of e-cigs on oral health is not fully understood and is still debated among many scientists and clinicians. The number of studies addressing the potential toxic effect of ENDS or e-cig aerosol on oral cells is limited along with the clinical studies which are still preliminary, and their sample size is limited. The long-term effects of inhaled aerosols and the potential synergistic effect of the e-cigs components are not known. It is essential and of utmost importance to determine whether exposure to ENDS aerosol contributes to the progression of periodontal diseases and how it affects periodontal ligament and gingival cells which are believed to be its first targets. This review briefly summarizes the available evidence about the effects of e-cigs on periodontal health including several pathophysiological events, such as oxidative stress, DNA damage, inflammation, cellular senescence, dysregulated repair, and periodontal diseases.

## 1. Periodontal Diseases and Tobacco Smoking

Periodontal diseases are multifactorial infections that are initiated by the interplay between the bacteria found in the dental plaque and the response of host immune response to this bacterial infection in the structures around the teeth (the gums, periodontal ligament, and alveolar bone). Bacterial infections drive an increased host immune-inflammatory response that causes swollen and bleeding gums, resulting in gingivitis (the earliest stage) and in loosening of the teeth (the advanced stage), the sign of severe periodontitis. Thus, periodontal diseases are characterized by chronic inflammation of the supporting tissues of the teeth [1,2].

Alterations in the local inflammatory cytokine profile are largely responsible for causing inflammation which is followed by increased bleeding on probing (BOP) and increased gingival crevicular fluid (GCF) flow. These local inflammatory cytokines may be found in GCF, saliva, and serum thus might serve as potential diagnostic or prognostic markers for the progression of periodontitis [3]. Interestingly, smokers exhibit less obvious gingival inflammation [4] and gingival bleeding [5,6] than nonsmokers, owing to the perturbed inflammatory response. In addition, gingival blood flow, BOP, and GCF flow increase as early as 3–5 days following smoking cessation [6,7].

Tobacco smoking is one of the most significant risk factors that influence the host immune-inflammatory response and periodontal diseases have been one of the most widely studied oral conditions in relation to cigarette smoking. There is an association between smoking and the loss of gingival attachment, the increase of gingival regression, tooth loss, deeper periodontal pockets, and more extensive alveolar bone loss along with the destruction of connective tissue and matrix [8,9,10,11,12]. Cigarette smoking is also one of the most important known contributors to the development of oral leukoplakia [13], and palatal leukokeratosis [14] but it can also modify the oral microenvironment so that several opportunistic pathologies may occur, such as oral candidiasis and hairy tongue [15]. Quitting smoking entails a decreased exposure to the risk of oral cancer and development of periodontal diseases [14,16].

Studies have shown that smokers have worse oral hygiene than nonsmokers and the smoking habit increases the mineralizing potential of saliva [17,18]. Between smokers and nonsmokers the plaque quantity, architecture, and bacterial composition of the teeth are rather comparable; although smokers exhibit a nicotine-related vasoconstriction of the gingival tissue leading to a slight decrease of the GCF flow that may lead to an impaired immunological response to bacterial growth on dental tissues (reviewed in [19]). Studies demonstrate a strong positive correlation between the use of tobacco products and the severity of periodontal disease.

## 2. Electronic Nicotine Delivery Systems (ENDS) Aerosol Constituents in Comparison to Those Found in Combustible Tobacco Smoke

Electronic nicotine delivery systems (ENDS; electronic cigarettes (e-cigs) and heat not burn devices (HnB)) have rapidly gained popularity as they were assumed to be a safer alternative to tobacco combustion, especially among younger individuals, pregnant smokers, and as tools for smoking cessation. 

ENDS, such as e-cigs and HnB products deliver stimulant nicotine to the users in aerosol state contributing to the chemical part of the addiction and at the same time they offer sensory and motor stimuli resembling smoking, but without the occurrence of the tobacco burning process. E-cigs are battery-powered nicotine-delivery devices without containing tobacco but using a liquid (e-liquid) that is vaporized to form nicotine-comprising aerosol, whereas HnB products contain tobacco that is directly or indirectly heated (but not burnt) using a variety of heat sources in order to create an inhalable tobacco aerosol. 

Although e-cigs were developed and marketed as a healthier alternative to smoking tobacco products, there is a growing body of evidence proving that their aerosols contain numerous toxicants, carcinogens, and organic compounds produced through thermal decomposition of the solvents, although their quantity is generally lower than the ones found in conventional tobacco cigarettes [20]. In studies comparing e-cigs aerosol to tobacco smoke, lower levels (9- to 450-fold lower) of potentially toxic compounds (e.g., formaldehyde, acetaldehyde, acrolein, and toluene) [21] but considerable levels of potential carcinogens including toxic metals (aluminum, cadmium, chromium, copper, lead, magnesium, manganese, nickel, and zinc), a number of organic compounds including carbonyls (e.g., acrolein from glycerol/glycerine), and potentially harmful compounds such as silicate beads, tin, and flavorings as well as propylene oxide (from propylene glycol) that are not present in traditional tobacco cigarettes are found in these aerosols [22,23,24,25,26,27,28,29,30]. Additionally, a general lack of standards in manufacturing and marketing of e-liquids has been reported [31]. Thus, a significant concern survives regarding varying purity, toxicity, and variety (e.g., flavor additives) of ingredients employed [32,33].

Besides the toxicants shared in tobacco smoke and e-cigs aerosols, diverse adverse effects were reported to be common, such as oxidative stress [34]. Similar to tobacco smoke, e-cigs aerosol includes reactive oxygen species (ROS) [34] that cause oxidative stress. Oxidative stress induced by ENDS or by the byproducts from their devices may be diverse and depend on a set of parameters, such as different volume of vapor, voltage settings, the type and state of the heating system and the type of atomizers, as well as the composition of e-liquids (humectant mixture, nicotine quantity, and addition of flavors, etc.). For example, aldehyde release in the aerosol of e-cigs varies depending on different power settings; high levels of aldehyde were detected in liquid overheating conditions which are higher voltage settings causing an unpleasant burn taste related to overheating of liquids whereas aldehyde emission was minimal under normal vaping conditions [35]. Aldehydes and free radicals found in e-cigs aerosols and tobacco smoke can cause oxidative stress, alterations in cell antioxidant activity, and they are also reported to trigger various types of DNA damage which can be repaired mainly by nucleotide excision repair (NER) and base excision repair (BER) mechanisms [34,36]. Furthermore, ROS/aldehydes/carbonyls derived from e-cigs aerosol can cause protein carbonylation, affecting auto-antibody production, which may lead to the destruction of the matrix and bone loss during periodontitis [37,38]. Methanol, propylene glycol, and glycerine have been shown to increase the generation of H_2_O_2_ [39]. Acrolein, a byproduct, has been reported to induce oxidative stress and inflammation resulting in loss of endothelial cell barrier integrity in the lung [40]. Thus, it is essential to investigate more on whether exposure to ENDS aerosol is a significant source of DNA damage in oral tissue, especially in periodontal/gingival cells in the oral cavity as these cells are directly exposed to ENDS vapor.

Many harmful constituents that are carcinogenic are still present in e-cig vapor, although at much lower levels compared to cigarette smoke. Endogenous formation of the tobacco-specific oral and esophageal carcinogen N’-nitrosonornicotine (NNN) were analyzed in 20 e-cig users, 20 smokers, and 19 nonsmokers. Analysis revealed that the mean of NNN in saliva of e-cig users was 14.6 (±23.1) pg/mL (ranging from nonquantifiable to 76.0 pg/mL) whereas in smokers, salivary NNN ranged from below the limit of quantification to 739 pg/mL, with 80% of smokers having salivary NNN in the range of levels found in e-cig users. These findings demonstrated that carcinogenic NNN was produced endogenously in e-cig users as well although the overall exposure to NNN in e-cig users is lower than in smokers [41].

In addition to all the toxic and potentially harmful components of ENDS aerosol, a majority of ENDS still contain the addictive drug nicotine, which is known to contribute to the development of cardiopulmonary diseases, neurodegenerative disorders, and cancer [36,42,43]. It is well-known that nicotine plays a significant role in the pathogenesis of cigarette smoking-associated diseases, such as periodontitis [44]. Nicotine is also shown to play a role in migration inhibition, cytoskeleton alterations, and extracellular matrix remodeling in human gingival fibroblasts (HGFs) [45,46,47] and it is reported to increase the amount of pro-inflammatory cytokines secreted in cultured gingival keratinocytes and fibroblasts [48,49]. Nicotine also was indicated as a contributing cause of periodontal degradation by affecting the attachment ability of the fibroblasts [50], as well as collagen and integrin production [51,52]. Considering that varying concentration of nicotine in commercial e-liquids may reach as high as 72 mg/mL (claimed on the label) [33], ENDS should be approached with caution as a risk of oral and general health.

## 3. In Vitro Studies on Effects of E-Cigs on Oral Cells and Tissues

In vitro studies have been emerging on the effects of e-cigs and their substances as their popularity continues to rise. Wisniewski and colleagues [53] studied the effect of liquid nicotine exposure on oral dysplastic keratinocytes and reported that nicotine triggers a migratory phenotype by activating EGFR signaling through a marked increase in fatty acid synthase (FASN) expression, a common pro-oncogenic event [54] which might also be relevant to oral carcinogenesis. In oral squamous cell carcinoma (OSCC), EGFR overexpression and its aberrant pro-oncogenic signaling are strongly associated with tumor progression in advanced clinical stages and worse survival rate outcomes [55,56]. Although liquid nicotine was tested and not vaporized nicotine derived from e-cigs, the study presents evidence for its role in FASN/EGFR signaling and increased migration of premalignant cells through EGFR signaling. This raises concerns about e-cigs safety, especially for former cigarette smokers with unknown oral premalignant lesions in which nicotine could trigger oncogenic signals associated with malignant progression [53].

The cytotoxicity of e-liquids; nicotine-containing or nicotine-free, direct or vaped, was investigated on the HGFs in order to assess the safety of these new electronic devices in the oral environment [57]. Oxidative stress was induced by both nicotine-containing and nicotine-free e-liquids with an increase in the expression of pro-apoptotic protein leading to the induction of early and late apoptosis. Oxidative stress generation was more pronounced for nicotine-containing liquid treated samples, as nicotine is known to contribute to the generation of intracellular oxidative stress [58], although also observed for samples treated with nicotine-free fluids compared to the untreated samples and so signifying that the e-liquids composition itself plays a role in increased Bax expression and in triggering of apoptosis in HGFs [57].

Similarly, Yu et al. reported that cells from different origins exposed to e-cig vapor extracts exposed presented significantly reduced viability and clonogenic survival, along with increased rates of apoptosis and necrosis in vitro, regardless of e-cig vapor nicotine content. They also exhibited significantly increased DNA strand breaks demonstrating increased comet tail length and accumulation of γ-H2AX foci [59].

Another study, repeatedly exposed HGFs to condensates of cigarette smoke and e-cig vapor (nicotine-rich or nicotine-free) for 60 min once a day for various time periods. Results of different analysis (MTT and BrdU assays) indicated that cells exposed to cigarette smoke or nicotine-rich condensates altered the morphology and reduced the proliferation rate. Compared to the controls, fibroblast cultures exposed to all condensates exhibited increased levels of TUNEL-positive apoptotic cells. The cell scratch test showed that repetitive exposures to cigarette or e-cig vapor condensates delayed both fibroblast migration and wound healing. Collectively, results represented that cigarette condensate was much more harmful to gingival fibroblast than e-cig vapor condensate, nicotine-free e-cig vapor being the least harmful [60].

Human organotypic buccal and gingival epithelial cultures established on transwells were exposed to tobacco smoke and aerosol generated by HnB tobacco product, matched by delivered nicotine doses. HnB aerosol-treated cells exhibited minor morphological changes overall in comparison to tobacco smoke-treated cells, and there were no signs of explicit cytotoxicity at even higher concentrations of HnB, whereas morphological changes and cytoskeleton reorganization were observed at the molecular level. The overview of differentially expressed genes and the biological interpretation of the data revealed that xenobiotic metabolism, oxidative stress response, and inflammation-related processes were consistently influenced by both the cigarette smoke and HnB aerosol treatments [61]. At comparable concentrations, tobacco smoke had higher impact on gene expression related to oxidative stress network than HnB aerosol, and also caused a greater effect on buccal samples compared to gingival samples. In analysis of miRNAs in buccal and gingival cultures, tobacco smoke treatment resulted in 265 and 264 differentially expressed miRNAs, respectively, whereas HnB aerosol-induced only four and 145 differentially expressed miRNAs. In summary, although substantial changes in mRNA, miRNA, and protein levels of structural molecules in cigarette smoke and HnB aerosol exposed buccal and gingival cultures were observed, HnB aerosol appears to have less effect on miRNA expression compared to cigarette smoke [61].

It is hypothesized that nicotine delivering e-cigs might impair healing of the bone/implant interface. It has been shown that smokers have an increased risk of dental implant failure and lower implant survival rates [62]. To determine the possible adverse effects of e-cig vapor on osteoblast interaction with dental implant material, osteoblasts were cultured onto titanium (Ti6Al4V) implant disks and exposed to whole cigarette smoke and to nicotine-rich or nicotine-free e-cig vapor for 15 or 30 min once a day for 1, 2, or 3 days. Osteoblast growth on the titanium implant disks was reduced significantly (*p* < 0.001) upon exposure to cigarette smoke as well as nicotine-rich and nicotine free e-cig vapors compared to nonexposed cells. The dysregulated attachment was shown to be due to decreased production of adhesion proteins such as F-actin, and due to reduced alkaline phosphatase (ALP) activity and tissue mineralization. Increased levels of caspase-3 protein following exposure of the osteoblasts to cigarette smoke or e-cig vapor was also responsible for the adverse effects on osteoblast dental implant material interaction. Collectively, although impairments in interaction of osteoblasts and titanium implant disks were observed upon exposure to e-cig vapor, the adverse effects of cigarette smoke on osteoblast growth, attachment, ALP, and mineralized degradation were greater than those of the nicotine-rich and nicotine free e-cig vapors [63].

Tobacco smoking is related to impaired healing, poor papilla regeneration, and increased bone loss [64,65]. High levels of nicotine have been shown to be antiproliferative and to cause toxic effects on osteoblast and bone metabolism whereas concentrations matching to light and moderate smoker yields increased osteoblast proliferation and bone metabolism [66]. In a clinical study, it was shown that free gingival graft donor-site wound healing was significantly altered due to reduced immediate bleeding incidence and delayed epithelialization in smokers [67]. Berley et al. reported a significantly reduced bone-to-implant contact in rat femurs that received subcutaneous nicotine [68]. Accordingly, Yamano et al. reported that bone matrix-related genes around implants were shown to be downregulated in rats that received nicotine for 8 weeks [69]. Collectively, although the effects of nicotine delivery by e-cigs on peri-implant soft and hard tissues as well as other periodontal complications have not been studied in detail yet, it is possible that nicotine derived from e-cig usage (vaping) may impair healing potential at the bone/implant interface.

Another consideration is the risks associated with ingestion of e-liquids although they are intended to be inhaled as aerosols. Vapor droplets may reach the oral mucosa or to the upper aerodigestive tract during the normal vaping session, or by accident [70], or intentionally in suicide attempts [71]. Direct exposure to e-liquids has been shown to produce harmful effects in periodontal ligament cells and gingival fibroblasts in culture [57,72].

A study examined 42 refill e-liquids for the presence of microorganisms, numerous chemicals, and solvents from 14 different brands in the market. It is reported that all the liquids complied with the norms for the absence of yeast, mold, aerobic microbes, *Staphylococcus aureus*, and *Pseudomonas aeruginosa*. They contained diethylene glycol, ethylene glycol, and ethanol within the authorized limits for food and pharmaceutical products. Terpenic compounds and aldehydes were also detected in the products, in particular, formaldehyde and acrolein, whereas no sample contained nitrosamines at levels above the limit of detection (1 μg/g) [33]. It was estimated, according to the lethal dose 50 (LD50) for various animals such as rodents and guinea pigs, that the risk of acute toxicity due to the components other than nicotine, with an ingestion of 10 mL of e-liquid, was not significant for humans. From the concentration of components reported, all the concentrations were at least 480 times (and 120 times for children) below the LD50 [33]. However, a minority of e-liquids, especially those with flavorings, incorporating particularly high varieties of chemicals, raises concerns about their potential toxicity in case of chronic oral exposure. This study considered hypothetical oral ingestion and interpreted the oral toxicity of detected compounds as ingested compounds that go through the first-pass metabolism, whereas inhaled compounds have direct access to the bloodstream without being metabolized, thus further studies which focus on vapors/aerosols of these liquids are needed as the inhaled form of e-liquids may include byproducts generated with the heating and vaporization processes that may exhibit different toxicity.

## 4. Studies on Oral and Periodontal Tissues

### 4.1. Studies on Direct Health Effects of ENDS in the Oral Cavity

There have been a few studies that address the direct health effect of e-cigs usage, especially regarding the oral cavity. In a cross-sectional analysis, it is reported that daily e-cig usage was associated with significantly increased odds of permanent loss of any tooth from nontraumatic causes in adults in the USA and it was indicated that vaping may be a risk factor for poor oral health outcomes including periodontal disease and tooth loss [73]. An association between e-cigs usage and higher odds of cracked/broken teeth, pain in the tongue and/or inside-cheek as compared to those who had never used e-cigs, among adolescents was previously shown [74].

In a study in which self-reported gingival disease among cigarette smokers and users of other types of tobacco products was evaluated, never users had the best periodontal health compared to users of various patterns of tobacco products [75]. Additionally, it is reported that the dual usage of e-cigs and conventional cigarettes among adolescents is related to poor oral health outcomes based on self-reported diagnosis while there is no significant association between past 30 days use of conventional cigarettes or e-cigarettes and past-year self-reported provider diagnosis with dental problems [76].

Clinical periodontal parameters (plaque index (PI), BOP, probing pocket depth (PPD), and clinical attachment loss (CAL)), radiographic (marginal bone loss (MBL)) and whole salivary cotinine, interleukin (IL)-1β, and IL-6 levels were investigated in a study that included 154 male individuals (39 cigarette-smokers, 40 waterpipe-smokers, 37 e-cig users, and 38 never-smokers). Collectively, clinical and radiographic parameters of periodontal inflammation were worse in cigarette and waterpipe smokers than e-cig users and nonsmokers. Whole salivary cotinine levels were similar in all groups while IL-1β and IL-6 levels in whole saliva were higher in cigarette- and waterpipe-smokers than e-cig users and never-smokers. Among e-cig users and never-smokers, there was no significant difference in the whole salivary IL-1β and IL-6 levels, in PPD, CAL, and mesial and distal MBL as well as in unstimulated whole salivary flow rate, whereas percentage of sites with plaque were significantly higher among e-cig users compared never-smokers [77].

Others also have shown that, as compared to smokers, e-cigs users and never-smokers have less periodontal inflammation and lower self-reported oral symptom scores [78,79]. Changes in the oral and general health status were assessed in a population of randomized smokers who have dropped cigarettes and started to use e-cigs. Periodontal health status, especially the plaque, and periodontal bleeding were analyzed and also a self-assessment questionnaire was included in order to evaluate the awareness of patients involved in this study about the changes in their general health status by switching from conventional cigarettes to e-cigs. From the beginning to the end of the observational period, a constant reduction of bacterial plaque on teeth surfaces as well as improvements for gingival bleeding were observed [79]. According to the self-assessment questionnaire, almost 71% of the subjects experienced an improvement in their general health status at the end of the observational study, less than 1/3 of all participants did not feel any clear difference, and only two subjects indicated a worsening. The majority (over 80%) of subjects clearly specified a positive variation in both smell and taste perception while the remaining minority did not feel substantial changes to disclose [79].

Another preliminary study, with a small sample of patients, evaluated the prevalence and characteristics of oral mucosal lesions (OMLs) in former smokers (*n* = 45) compared to e-cigs consumers (*n* = 45). The prevalence of OMLs was higher among e-cigs users (65.4%), compared to the former smokers (34.6%) although the difference between the two groups was not statistically significant in terms of total prevalence of OMLs. Furthermore, nicotine stomatitis, hairy tongue, and hyperplastic candidiasis in the retro-commissural area occurred in a greater frequency among e-cig users than in former smokers [80]. 

To evaluate the prevalence of cellular changes in the oral mucosa in traditional cigarette smokers and e-cigs users, in comparison with nonsmokers, scrapings of the oral mucosa from the three groups of participants (smokers, e-cig smokers, and nonsmokers) were cytologically examined using micronucleus assay test which is a cytological method that has been used to assess OSCC risk in smokers or generally, in subjects exposed to carcinogens [81]. The prevalence of micronuclei in oral cavity cells exhibited a statistically significant decrease in e-cigs users similar to those of controls, compared to that in the smokers group, based on the average total number of micronucleated cells/1000 cells and the average total number of micronuclei/1000 cells values. Their results demonstrated that there were no statistically significant alterations in the micronuclei distribution among e-cigs users [81].

Gingival health was also evaluated in a pilot study in which a group of established smokers was examined before and after substituting vaping instead of smoking tobacco. When smokers switched from cigarettes to e-cigs for 2 weeks, there was a statistically significant increase in gingival inflammation, percentage of sites with BOP; similar direction to that which occurs when smokers quit, and an increase in GCF volume, an alternative parameter which also reflects gingival inflam-mation whereas the levels of plaque were similar between visits [82]. Previously, reduction in bleeding was suggested to be due to induction in gingival vasoconstriction caused by nicotine [83], however, based on this study, it is unlikely that nicotine is the sole causing reagent for gingival vaso-constriction and reduction in BOP since both cigarettes and e-cigs provide a source of nicotine.

### 4.2. Studies on Other Effects of ENDS in the Oral Cavity

With the aim to characterize the effects of e-cigs aerosol and tobacco smoke exposure on the bacterial profiles at multiple distinct and relevant body sites, a study examined cross-sectional oral (saliva, buccal swabs) and fecal samples from a human cohort consisting of 30 individuals (10 e-cigs users, 10 tobacco smokers, and 10 controls). E-cigs users had no effect on the oral or gut communities whereas tobacco smoking had a significant effect on the bacterial profiles in all sample types when compared to controls and to the e-cigs group [84], although this study remains limited in sample size and duration to validate a clear association. On the other hand, a cross-sectional study comparing oral Candida carriage among cigarette- (*n* = 34) and waterpipe-smokers (*n* = 33), e-cig users (*n* = 30), and never-smokers (*n* = 32) reported that oral Candida carriage rates were 100%, 100%, 83.3%, and 50%, respectively. *Candida albicans* was the most commonly isolated oral yeast species in all groups and *C. albicans* carriage was significantly higher in cigarette smokers (*p* < 0.05), waterpipe-smokers (*p* < 0.05), and e-cig users (*p* < 0.05) than never-smokers [85]. An in vitro study showed that nicotine-rich e-cigs vapor exposure had a positive impact on *C. albicans* growth compared with nonexposed cultures [86]. Following exposure to e-cig vapor, *C. albicans* produced high levels of chitin and exhibited increased hyphal length and the expression of different virulent genes such as *SAP2*, *SAP3*, and *SAP9*, which are known to contribute to *C. albicans* growth and virulence. E-cig vapor exposed *C. albicans* adhered better to epithelial cells than the control when they were in contact with gingival epithelial cells whereas indirect contact between e-cig-exposed *C. albicans* and gingival epithelial cells caused epithelial cell differentiation, reduced cell growth, and increased lactate dehydrogenase activity. Overall, results indicate that e-cig may interact with *C. albicans* to promote their pathogenesis, which may increase the risk of oral candidiasis in e-cig users [86].

Deregulation in critically important genes, molecular pathways, and functional networks in the oral epithelium may be related to cancer. The regulation of genes and associated molecular pathways, genome-wide, were compared in oral cells of e-cig users and cigarette smokers to nonsmokers by RNA-sequencing (RNA-seq). Interrogation of oral transcriptome exhibited deregulation of important genes and associated molecular pathways in oral epithelium of e-cig users that both resemble and differ to that of smokers. Analysis showed a significant number of aberrantly expressed transcripts in both e-cig users and smokers relative to nonsmokers whereas cigarette smokers had ~50% more differentially expressed transcripts than e-cig users (1726 versus 1152). The “Wnt/Ca^+^ pathway” in e-cig users and the “integrin signaling pathway” in smokers were the most affected pathways in the canonical pathways and networks modulated. For both e-cig users and smokers, the “Rho family GTPases signaling pathway” was the top disrupted pathway amongst the overlapping functional pathways, although the number of affected targets was three times higher in smokers than e-cig users [87].

In a small study which included 10 volunteers, capillary blood flow in the buccal mucosa was measured with 5-min intervals using a laser Doppler probe after vaping nicotine-free or nicotine-containing e-liquid for 5 min. A wide variation was observed in the results, however a small but significant rise of the blood flow was observed as a consequence of vaping, thus indicating that e-cigs usage may have an effect on blood flow to the oral mucosa [88]. However, the measurements were not performed in smokers and no further comparison could be made.

In addition to the potential and known harmful effects of tobacco consumption or e-cigs usage on oral tissue, there have been injury cases reported related to explosions [89]. E-cigs explosions are mostly related to the battery malfunction/quality, e-cig device design, or compatibility between the device and the charger. Injuries caused by e-cigs explosions can be due to flame burns, chemical burns, or blast injuries and may result in abdominal burns, oral lacerations, teeth fractures, and avulsions. The nature and circumstances of the injuries suggest that these incidents were unintentional and they would potentially be prevented through battery design requirements, testing standards, and public education related to ENDS battery safety [90].

## 5. Concluding Remarks

ENDS are relatively new devices with increasing popularity each day. There is very little known regarding the effects of ENDS aerosol on health and there is also little evidence on their long- or short-term effects on oral health. It is well-known that cigarette smoking produces a major risk for periodontal and likely for peri-implant diseases. Although e-cigs may be less harmful than traditional smoking, they can still contribute to the pathogenesis of periodontal diseases by inflammation, cell injury, and impaired reparability. E-cigs vapor, with or without nicotine, and its additional flavoring agents may harm periodontal ligament, stem cells, and gingival fibroblasts in cultures due to the presence of aldehydes/carbonyls that lead to protein carbonylation of extracellular matrix, DNA adducts/damage, and cellular senescence. We have summarized the currently known effects of e-cigs on oral cells and health in the blue box below. As with results from all in vitro studies, the implications of these studies for human health are not so clear. Preliminary clinical studies are emerging; however, they have not been long-lasting yet and they have remained restricted to a small sample of participants, something that limits their validity. All these things considered, more research is needed to clarify the implications of e-cigs usage on oral health, particularly when compared with smoking, and this is an important for future studies. 


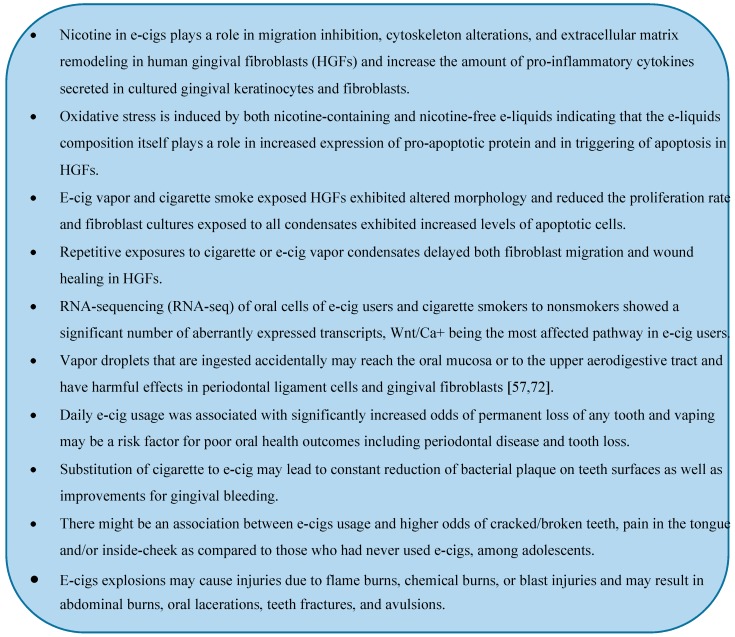


## 6. Methods

**Figure 1:**
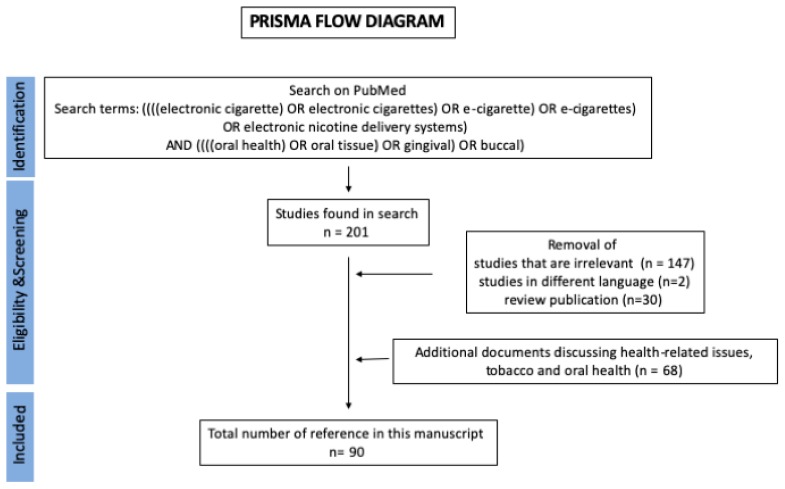
Prisma flow diagram showing the methodology for literature review and selection of studies.

For this review, we searched the PubMed electronic database for English language articles by using keywords related to e-cigs and/or their combination (e-cigarette, electronic cigarette, electronic nicotine delivery systems (ENDS), and oral health) without any date restriction, we did not include HNB in the search criteria. The Prisma Flow Diagram for the search is shown in Figure 1. We obtained a total of 201 results. After careful review of the titles, abstracts, and full text, 179 studies were excluded and we judged 22 studies to be relevant to research on e-cigs and oral health. Reference lists from these studies were also examined to identify relevant articles and we searched additional information that were available online. The current review presents the findings from 22 published studies and in total 90 studies are cited.

## Abbreviations

BER	Base Excision Repair Mechanisms
BOP	Bleeding on Probing
e-cigs	Electronic Cigarettes
e-liquid	E-cig Liquid
ENDS	Electronic Nicotine Delivery Systems
GCF	Gingival Crevicular Fluid
HGF	Human Gingival Fibroblasts
HnB	Heat not Burn
NNN	N’-nitrosonornicotine
OMLs	Oral Mucosal Lesions
OSCC	Oral Squamous Cell Carcinoma
ROS	Reactive Oxygen Species
